# Smartphone application for wound area measurement in clinical practice

**DOI:** 10.1016/j.jvscit.2021.02.008

**Published:** 2021-02-26

**Authors:** Rodrigo Bruno Biagioni, Bruno Vinicius Carvalho, Renato Manzioni, Marcelo Fernando Matielo, Francisco Cardoso Brochado Neto, Roberto Sacilotto

**Affiliations:** Division of Vascular Surgery, Hospital do Servidor Público Estadual, São Paulo, SP, Brazil

**Keywords:** Application, Area, Measurement, Point-of-care, Smartphone, Wound

## Abstract

A total of 85 consecutive patients had their wound area measured. The procedure was executed in two parts. The first was to take photographs of the wound using a smartphone and measure the area using the imitoMeasure application (imito; imito AG, Zurich, Switzerland) by two raters. The second was to take photographs of the same wound using a 10-megapixel digital camera and posterior measurement of the area using ImageJ software (National Institutes of Health, Bethesda, Md) by one operator. The mean area of the wounds was 12.20 ± 10.45 cm^2^ for imito and 12.67 ± 10.86 cm^2^ for ImageJ measurement. The interclass correlation coefficient (ICC) between ImageJ and imito was 0.978 for a single measure and 0.989 for the average measure. Considering the two measurements, the ICC demonstrated excellent interobserver correlation using imito (0.987). Larger wounds had a greater difference between the methods (4.28% greater with the ImageJ measurement when considering areas >9 cm^2^). No difference was found between iOS (ICC, 0.995) and android (ICC, 0.970) smartphone operating systems. The smartphone application is a useful method for area measurement with excellent accuracy compared with digital photography and the ImageJ processing tool.

Area measurement has been recommended for venous and arterial wounds.^1^, ^2^, ^3^ Only 5% of clinical trials involving wounds have referred to the validity or reliability of the measurement methods used.^4^ Planimetry,^5^ digital photography followed by computer software program analysis,^5^^,^^6^ direct measurement of two diameters of the wound (ellipse area calculation),^7^^,^^8^ dedicated photography software (using a smartphone application [app]),^9^ and laser technology^6^ have been the main methods used in clinical trials.^7^ Although many methods are available, the most commonly used have been computer software programs and planimetry.^4^

The ideal method would be one that simultaneously offers accuracy, reliability (repeatability), and feasibility.^7^ The use of a smartphone app promotes good feasibility when used as a point-of-care tool.^9^ Comparisons with a previously validated method will ensure reliability^5^^,^^7^ and interrater agreement.^9^

The present study compared smartphone applications to photography and ImageJ software (National Institutes of Health, Bethesda, Md) program measurements regarding the accuracy and reliability in real-world clinical practice.

## Methods

A total of 85 consecutive patients had had their wound area measured from February 2017 to March 2019. The patients were recruited from the inpatient and outpatient clinics of a vascular surgery department. All the patients provided written informed consent, and the local committee approved the research protocol.

The inclusion criterion was a wound on the foot or leg caused by vascular disease. The exclusion criterion was a wound with a circumferential shape without the possibility of two-dimensional area measurement. The demographic aspects, localization, and etiology of the wounds were registered prospectively using a dedicated protocol.

The measurement procedure consisted of two parts. The first was to take photographs of the wound using a smartphone and measurements using the imitoMeasure application (imito; imito AG, Zurich, Switzerland). The second was to take photographs of the same wound using a Canon 10-megapixel camera (Canon, Tokyo, Japan) and posterior measurement of the area using ImageJ software (National Institutes of Health, Bethesda, Md).

**Fig 1:**
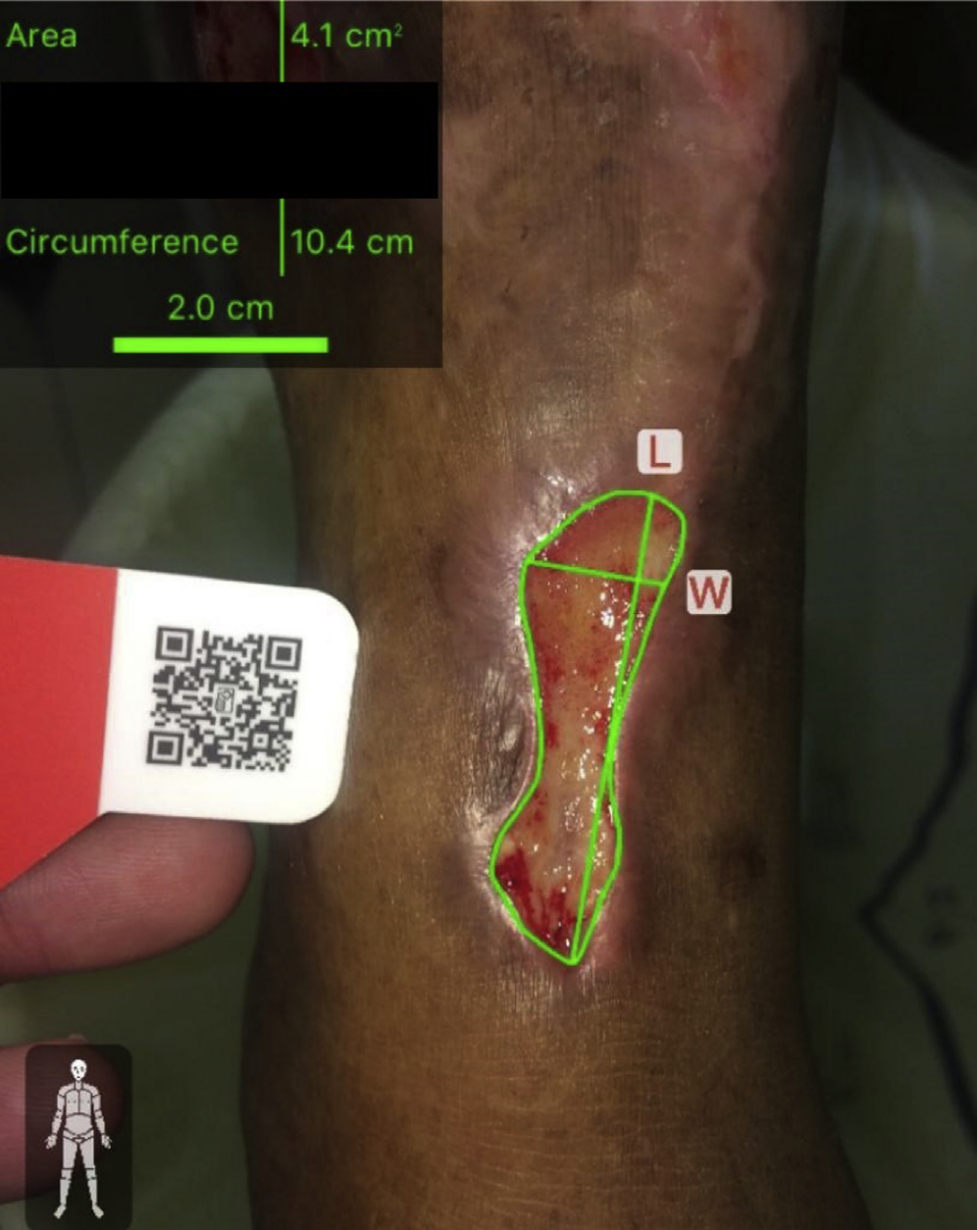
Photograph of microangiopathic wound at the anterior leg with the area measurement using the imitoMeasure (imito) application (app).

First, the segment of the leg was selected in the imito app. The camera of the smartphone was positioned ∼20 to 30 cm away from and parallel to the wound. The calibration marker (quick response [QR] code) was positioned next to and in the same plane of the wound, and a photograph was taken after recognition of the QR code by the imito app. The operator's finger was used to select the borders of the wound through the photograph of the wound. The imito app reported the results of the area, width, height, and circumference (Fig 1). This procedure was performed by two different operators (vascular surgeon staff or residents with previous basic training) and using two independent photographs within a few minutes. The iOS operation system (iPhone 6S; Apple, Cupertino, Calif) was used for 42 patients, and the android operation system (Samsung Galaxy S8; Samsung, Seoul, Korea) was used for 43 patients.

**Fig 2:**
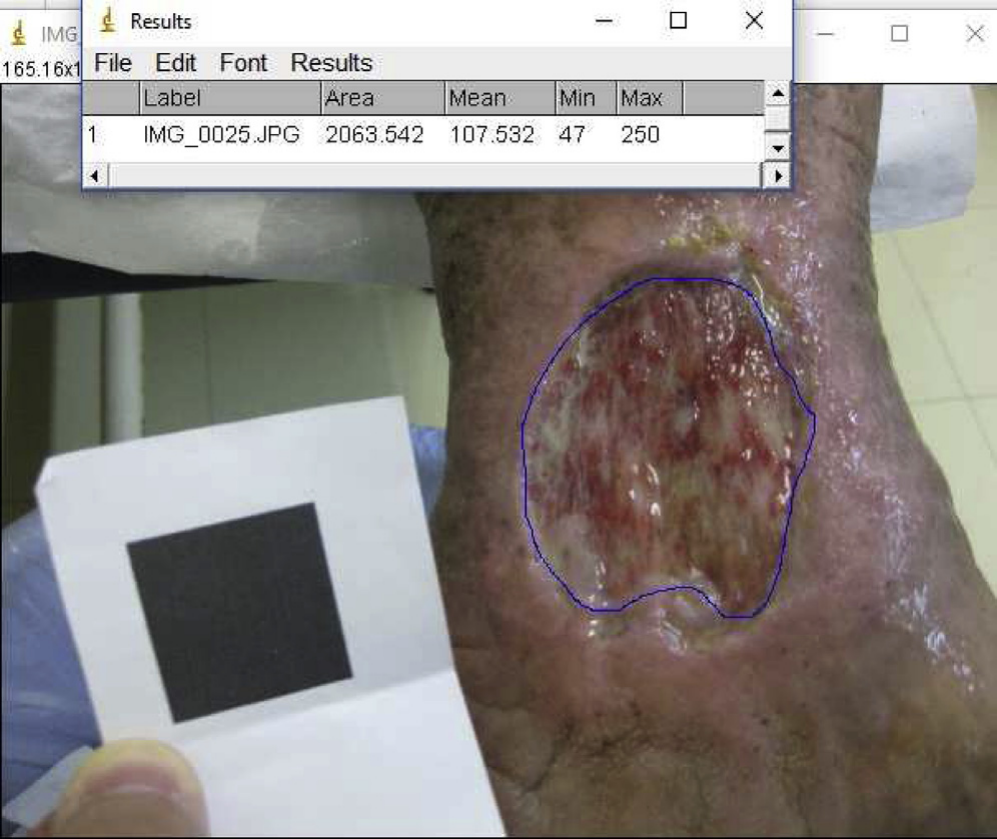
Photograph of the venous wound of the medial malleolus and posterior area measurement using ImageJ software.

In the second part, another photograph of the wound side by side with a black square measuring 3 × 3 cm was taken using the 10-megapixel digital camera. In the background, the image in JPEG format was analyzed using ImageJ software. Calibration was performed with the black square, and the measurement of the area was performed two times by the same operator (Fig 2).

All analyses were conducted using SPSS, version 20.0, for Windows (IBM Corp, Armonk, NY). A previous descriptive analysis was performed on the prevalence of the risk factors and wound localization and etiology. The comparison of the mean between the groups was executed using the Student *t* test. The interclass correlation coefficient (ICC) was computed, comparing the results between the two operators using the imito app (interrater analysis) and between the imito app and ImageJ software (accuracy). For ImageJ, the same observer analyzed the same image twice, and the area measurements were performed consecutively. A very high (excellent) correlation was considered present when the ICC was >0.9.^10^ *P* values <.05 were considered statistically significant.

## Results

Most of the patients were men (70.0%), and the mean age was 66.0 ± 14.7 years. For 64.8% of the patients, the wound was on the left side of the leg. The most frequent wound location was the toe (24%; Table I). The wound etiologies were arterial (ischemic; 43.3%), infectious (38.5%), venous (9.7%), and microangiopathic (8.5%). The mean area of the wounds was 12.20 ± 10.45 cm^2^ for imito and 12.67 ± 10.86 cm^2^ for ImageJ. The difference between the mean area measured using ImageJ and imito was 0.47 cm^2^ (3.71% greater using ImageJ). According to the *t* test (*P* = .121), no significant difference was present between the two groups. The ICC between ImageJ and imito was 0.978 for a single measure and 0.989 for the average measure. The 95% confidence interval (CI) was 0.983 to 0.993 (*P* < .0001; Table II).

**Table I:** Location of the wounds measured using imitoMeasure and ImageJ

Wound Location	No. (%)
Toes (including amputation stump)	22 (25.8)
Leg	17 (20.0)
Malleolar (medial or lateral)	14 (16.4)
Foot (dorsal)	12 (14.1)
Foot (plantar)	7 (8.2)
Transmetatarsal amputation	6 (7.0)
Heel	5 (5.8)
Transtibial amputation stump	2 (2.5)

**Table II:** Comparison of results between measurement tools

Comparison	*P* value (*t* test)	ICC
imito vs ImageJ	.121	0.978
imito vs imito	.847	0.987
ImageJ vs ImageJ	.480	0.999

*ICC,* Interclass correlation coefficient.

The mean areas by the same observer using imito were 12.23 ± 10.7 cm^2^ and 12.28 ± 10.91 cm^2^. Comparing these mean values, statistically, no difference was identified (*t* test; *P* = .847), and ICC demonstrated excellent interobserver correlation using imito (ICC, 0.987; 95% CI, 0.878-0.999). The mean areas of the two measurements with ImageJ were 12.65 ± 10.89 cm^2^ and 12.68 ± 11.07 cm^2^. No difference between the mean value was identified using the *t* test (*P* = .480), and the ICC was 0.999 (95% CI, 0.999-1.000). Both measurements by the same observer using the ImageJ method had excellent correlation (Table II). When analyzing the differences in the measurements of the different areas, a cutoff of 9 cm^2^ revealed a major difference between the two methods (4.28% and 1.17% greater using ImageJ for the images with areas superior and inferior to 9 cm^2^, respectively).

Considering the use of different smartphone operating systems, the areas were not different between imito and ImageJ. The ICCs comparing the results were 0.995 for iOS (95% CI, 0.991-0.997) and 0.970 for android (95% CI, 0.946-0.984). Nevertheless, the difference between the measurements was greater for the android than for the iOS system. For iOS, the difference was 1.15% between ImageJ and imito (*P* = .357) and 5.60% for android (*P* = .084).

## Discussion

Smartphones incorporating high-definition digital cameras are now widely available at a relatively low cost.^9^^,^^11^^,^^12^ The high portability and mobility provided by such devices are especially appealing for clinical application.^12^ Imito and other smartphone-dedicated applications have emerged to make wound measurement and documentation easier and simpler.^6^^,^^8^

The imito app is a noncontact digital planimetry application, providing an advantage compared with other methods. In the present study, the interrater differences were not significantly identified. Considering that all measurements are predicated on adequate photography and calibration positioning, some points must be considered when photographing the wound. First, the QR code must be positioned at the same level as the wound. This approach avoids underestimation or overestimation of the wound area. Second, the photographs must be taken directly of the wound, avoiding axis deviation. In another study, a deviation of 20° of the optical axis of the wound was found to lead to an underestimation of the surface by ∼10%.^13^ Third, the image must be positioned and sized in the smartphone screen to occupy the entire surface. The manual setting of the area in the imito app is obtained by tracing the circumference with a point-to-point line. With the amplified image, the distance between the points will be smaller, which improves the outline of the wound border. Considering such orientation, in another study, the investigators observed that professional medical photographers and relatively untrained clinician photographers did not differ in the area measurement.^14^

Two planimeter methods could be considered the reference standard for area measurement^15^: manual (Visitrak; Smith & Nephew Wound Management, Inc, Largo, Fla)^5^^,^^6^^,^^15^ and digital (Verg; Vista Medical Ltd, Winnipeg, Manitoba, Canada).^15^ They are widely used. However, they are expensive, require direct contact with the wound, and are not available in Brazil. Digital photography with ImageJ software processing has been the method used in our center and has already been validated with excellent correlation compared with the Visitrak results.^5^

Considering the results of the imito and ImageJ area measurements, the correlation was excellent, demonstrating the excellent accuracy of the smartphone application. The 3.76% underestimation of the imito measurements could be related to the point-to-point manual tracing of the wound outline.^6^ When the interrater ICC results were analyzed, an excellent correlation between the measurements was identified, even considering that the operators were different and had received little training. The two measures of imito had a lower ICC compared with those using ImageJ.

Despite the promising results, the method has some critical limitations. One of the limitations is the impossibility of area measurement of three-dimensional wounds. In particular, circumferential wounds of the leg and wounds after transmetatarsal amputation of one to three toes will have two or more planes required for complete evaluation of the wound surface. Another limitation is the impossibility of analyzing the depth of the wound. Some systems such as MAVIS (measurement of area and volume instrument system),^13^^,^^15^ MEDPHOS (medical digital photogrammetric system),^15^ and SilhouetteMobile (Aranz Medical Ltd, Auckland, New Zealand)^6^^,^^7^^,^^16^ have the appropriate technology. However, they are expensive and not promptly available for point-of-care, as are smartphone applications.^13^

## Conclusion

The imitoMeasure app is a useful and practical method for area measurement with excellent repeatability and accuracy compared with digital photography and the ImageJ processing tool.
